# Evaluation of drug-resistant tuberculosis treatment outcome in Limpopo province, South Africa

**DOI:** 10.4102/phcfm.v15i1.3764

**Published:** 2023-07-31

**Authors:** Ngwanamohuba M. Seloma, Marema E. Makgatho, Eric Maimela

**Affiliations:** 1 Department of Pathology and Medical Sciences, Faculty of Health Sciences, University of Limpopo, Polokwane, South Africa; 2 Department of Public Health and Health Promotion, Faculty of Health Sciences, University of Limpopo, Polokwane, South Africa

**Keywords:** tuberculosis, drug-resistant tuberculosis, treatment outcome, treatment success rate, End TB strategy

## Abstract

**Background:**

South Africa has the second-highest tuberculosis (TB) incidence globally. Drug-resistant TB (DR-TB) treatment has less successful treatment outcomes as compared with susceptible TB, and it hinders TB control and management programmes.

**Aim:**

This study aimed to evaluate drug-resistant TB treatment outcomes and factors associated with successful treatment outcomes.

**Setting:**

The study was conducted in five districts in Limpopo province.

**Methods:**

The study design was retrospective and descriptive. Patients’ demographic data, data on clinical characteristics and treatment outcomes data were extracted from the electronic drug-resistant tuberculosis register (EDRWeb) database system for the period, 2010–2018, in Limpopo province. Frequency, percentages and bivariate and multivariate logistic regression were used to analyse data using Statistical Package for Social Sciences version 27.0. The significance difference was determined at a 95% confidence interval and *p* < 0.05.

**Results:**

A total of 385 drug-resistant records were included in this study. The treatment success rate was 223 (57.9%). A total of 197 (51.2%) patients were cured, 26 (6.8%) completed treatment, 19 (4.9%) treatment failure, 62 (16.1%) died, 78 (20.6%) were recorded as the loss to follow-up, 1 (0.3%) moved to another country and 2 (0.5%) were transferred out.

**Conclusion:**

The treatment success rate was 57.9%, which is still below targets set by National Strategic Plan in South Africa and World Health Organization End TB targets.

**Contribution:**

The findings of the study reveal that to achieve successful DR-TB control programme and attain End TB targets, monitoring of treatment outcomes is crucial.

## Introduction

Tuberculosis (TB) is a chronic, curable, and preventable infection disease that continues to be a major cause of death in many developing countries.^1,2^ *Mycobacterium tuberculosis* causes TB, which is airborne and transmitted by infected droplets.^3,4,5^ Despite the availability of extremely effective treatment for decades, TB remains a major concern, especially in developing countries, which continues to be a problem, particularly in developing countries.^6,7^ The World Health Organization (WHO) estimates that 10 million people were affected by TB in the world in 2019, with 98% of cases occurring in developing countries.^5^ Africa alone reported the highest TB infections, accounting for 24%^5,8,9,10^ while South Africa accounted for 3.3% of the global TB burden in 2019.^11,12,13,14^

*Mycobacterium tuberculosis* strains that are resistant to one or more anti-TB drugs are referred to as drug-resistant TB (DR-TB).^1,2^ Treatment of drug-resistant TB, which is usually less effective than the treatment of drug-susceptible TB, is a major challenge for TB control and elimination programmes.^15,16^ To minimise transmission, and prevent drug resistance, timely and effectively, TB treatment is essential.^17,18^ Therefore, monitoring DR-TB treatment outcomes is critical when evaluating TB control strategies. Furthermore, monitoring treatment outcomes allows for comparisons across different geographic areas.^6^

Previous research shows that several factors contribute to poor DR-TB treatment outcomes. Comorbidity, previous TB treatment, older age, TB type, TB and human immunodeficiency virus (HIV) comorbidity, low income, limited access to transportation, distance from home to treatment facilities centre, limited interest in disease and treatment information, a lack of social support and poor adherence to treatment, have all been linked to unsuccessful treatment outcomes.^19,20,21^ Poor adherence has serious public health repercussions, including increased transmission and the emergence of drug resistance, and can result in relapse and even deaths of the patients. Therefore, the WHO advises that treatment outcome analyses, particularly among patients with pulmonary TB (PTB), should be conducted annually at the national and district levels.^22^

Tuberculosis treatment outcomes in South Africa were assessed in limited settings and the success rate varied accordingly based on different determinants.^23,24,25^ The National Strategic Plan developed an integrated treatment for TB, HIV and sexually transmitted infections (STIs). This entailed the development of new drugs and molecular diagnostic techniques. In 2011, the new management of DR-TB policy guidelines was approved, the decentralised management of multidrug resistant TB (MDR-TB) was introduced, followed by individualised treatment with second-line drug susceptibility results.^26,27,28^ The End TB strategy aims to reduce TB incidence by 90% and deaths to 95% in 2035.^11^ One way of assessing the effectiveness of the End TB Strategy, is monitoring of successful treatment outcome rate measured by the proportion of patients who are cured and have completed their treatment.^18^ Data on the effectiveness of this strategy are limited in the Limpopo province. Evaluating drug-resistant TB treatment outcomes and factors associated with successful treatment outcomes may benefit TB control and management programmes.^5,8,9^ The study aimed to evaluate drug-resistant TB treatment outcomes and factors associated with successful treatment outcomes among patients in the Limpopo province, South Africa.

## Research methods and design

### Study design

The study design was retrospective and descriptive. Drug-resistant TB patients data, in Limpopo province from 2010 to 2018, were evaluated.

### Study setting

The study was conducted in the Limpopo province, which is the northernmost province of South Africa (SA). It is bordered to the north by Zimbabwe, east by Mozambique, south by Mpumalanga, Northwest and Gauteng provinces of South Africa and northwest by Botswana. Limpopo province is divided into five districts, namely Waterberg, Capricorn, Mopani, Vhembe and Sekhukhune, for local municipality purposes. According to Statistics SA (2019) mid-year estimates, the province had a population of 5 982 584.^29^ In Limpopo, the rural portion of the population makes up about 80% of the total. Immigration to the province is prevalent, both legally and illegally. Based on the 2016 Health System Trust report, the province had many patients initiating treatment.^30^

### Study population

A total of 4031 drug-resistant TB patients registered from 2010 to 2018 in the Limpopo province were considered for data collection purposes. The files were accessed from the Department of Health in the Limpopo province on the EDRWeb system from the period 2010 to 2018. The province provides a TB control programme through a decentralised network of primary healthcare medical facilities that also offer other general health services at the district level. This study reviewed the records of 4031 patients enrolled in the EDRWeb system from 2010 to 2018.

### Sampling method

In this study, drug-resistant TB data from 2010 to 2018 in the Limpopo province were collected from the EDRWeb database using systematic random sampling.^31^ The study sampled all types of drug-resistant TB patients registered for treatment from 2010 to 2018 in the Limpopo province. Patients with documented treatment outcomes were included in this study. Patients’ files with incomplete data and incomplete treatment outcomes were excluded from the study. A total of 2916 patients’ drug-resistant files were randomised using Microsoft excel. Assuming confidence of 95%, a margin of error of 5% and a conservative prevalence estimate of 50%, a total sample size of 385 files was used in the study.

### Data collection

Demographic and clinical characteristics, TB treatment and treatment outcomes were collected from the EDRWeb database system from 2010 to 2018 in the Limpopo province. A total of 385 files were included in this study. The review considered socio-demographic factors, such as age, gender, districts, and clinical characteristics data. The clinical characteristic data encapsulated 6-month interval sputum results, registration type, previous DR-TB episode, previous drug history, patient treatment regimen, patient treatment category, pulmonary or extra-pulmonary, types of drug resistance and final treatment outcome. Treatment outcomes were classified according to the WHO’s recommendations.^32^

### Data analysis

Data were checked for completeness, and thereafter coded and entered into a Microsoft Excel spreadsheet. The Statistical Package for Social Science (SPSS) Version 27.0 software was used to analyse the data. Patients’ demographic and clinical characteristic data were summarised into frequency and percentage using descriptive statistics. Bivariate and multivariate logistic regression were used to analyse factors associated with successful treatment outcomes. Statistical significance was determined at a *p*-value of less than 0.05. The dependent variable was the TB treatment outcome. This variable meant successful treatment (cured, treatment completed), further defined as TB patients who completed treatment with a resolution of symptoms or were cured (negative smear microscopy at the completion of the treatment and on at least one prior follow-up test). The independent variables were 6-month interval treatment outcomes, which included the registration year, registration type, districts, previous drug history, patients’ category, type of TB, age and gender.

### Ethical considerations

The University of Limpopo Turfloop’s Research Ethics Committee (TREC) granted approval and ethical clearance for the study; project reference number: (TREC/180/2020: PG). Permission to conduct the study in Limpopo province was obtained from the Limpopo Provincial Department of Health (Ref: LP-2020-10-008).

## Results

There were more male patients (*n* = 215, 55.8%) than female patients (*n* = 170, 44.2%) in the study. The patients aged 20–39 were recorded as 48.1%, followed by those aged 40–59 with 40.3%. Those above the age of 60 comprised 6.5%, whereas 0–19 constituted 5.2% (Table 1). The Waterberg District had more patients (*n* = 304, 79%), followed by Capricorn, Vhembe, Mopani and Sekhukhune, respectively. The majority of DR-TB patients were diagnosed in 2013 and the least in 2010 (Table 1).

**TABLE 1:** Socio-demographic characteristics of drug-resistant tuberculosis patients from 2010 to 2018 (*N* = 385).

Variables	*n*	%
**Gender**		
Female	170	44.2
Male	215	55.8
**Age group (years)**		
0–19	20	5.2
20–39	185	48.1
40–59	155	40.3
60	25	6.5
**Districts**		
Mopani	13	3.4
Sekhukhune	11	2.9
Vhembe	22	5.7
Waterberg	304	79.0
Capricorn	35	9.1
**Patients’ registration year**		
2010	10	2.6
2011	21	5.5
2012	39	10.1
2013	66	17.1
2014	53	13.8
2015	55	14.3
2016	53	13.8
2017	38	9.9
2018	50	13.0

Table 2 shows the clinical characteristics of drug-resistant TB patients from 2010 to 2018. The final DR-TB treatment outcome results recorded for the patients cured (*n* = 197, 51.2%), treatment completed (*n* = 26, 6.8%) and (*n* = 78, 20.6%) those identified as the loss to follow-up; those who died (*n* = 62, 16.1%), those who moved out (*n* = 1, 0.3%), transferred out (*n* = 2, 0.5%) and those who experienced treatment failure (*n* = 19, 4.9%) (Table 2).

**TABLE 2:** Clinical characteristics of drug-resistant tuberculosis patients from 2010 to 2018 (*N* = 385).

Variables	Frequency	Percentage
**Outcomes at 6 months of DR-TB treatment**.		
Died	34	8.8
Failed	2	0.5
Loss to follow-up	39	10.1
Positive (smear or culture positive)	18	4.7
Negative (2 consecutive cultures)	286	74.3
Transferred out	6	1.6
**Registration type**		
Moved in	4	1.0
Transfer in	14	3.6
New	367	95.3
**Previous drug history**		
PT1 (Patient treatment regimen 1)	175	45.4
PT2 (Patient treatment regimen 2)	36	9.4
New TB cases	174	45.2
**Patient category**		
Other	8	2.1
Relapse	84	21.8
TF1	60	15.6
TF2	25	6.5
Re-treatment (Treatment after loss to follow-up)	34	8.8
New cases	174	45.2
**Pulmonary or extra-pulmonary**		
Extra pulmonary	2	0.5
Pulmonary	383	99.5
**Types of drug-resistant TB**		
MDR Lab confirmed	130	33.8
Mono	24	6.2
None	1	0.3
Poly	7	1.8
Pre XDR	8	2.1
Rifampicin	208	54.0
XDR Lab confirmed	6	1.6
XDR not Lab	1	0.03
**Final treatment outcome**		
Died	62	16.1
Moved out	1	0.3
Loss to follow-up	78	20.3
Transferred out	2	0.5
Treatment completed	26	6.8
Treatment failure	19	4.9
Cured	197	51.2

PT1, patient treatment regimen 1; PT2, Patient treatment regimen 2; TF1, Treatment failure after regimen 1; TF2, Treatment failure regimen 2; TB, tuberculosis; MDR, multidrug resistant; XDR, extensive drug-resistant; TB, tuberculosis; DR-TB, drug resistant tuberculosis.

Factors associated with successful treatment outcomes were determined using logistic regression (Table 3). Negative sputum conversion results at 6-month intervals (two consecutive cultures), registration year and female gender had a significant association with successful treatment outcomes. Registration types, districts, previous DR-TB episodes, previous drug history, patient category, pulmonary or extra-pulmonary, types of drug resistant TB, age, and patient category had no significant associations with successful treatment outcome (Table 3).

**TABLE 3:** Bivariate and multivariate logistic regression analysis factors associated with successful tuberculosis treatment outcomes in Limpopo province from 2010 to 2018.

Variables	COR	95% Cl	*p*-value	AOR	95% Cl	*p*-value
**Outcomes at 6 months of treatment**						
Positive (smear or culture positive)	Ref.	-	-	-	-	-
Died	0.0	0.0	0.996	2.2	0.0	0.991
Loss to follow-up	0.0	0.0	0.997	1098	0.0	0.994
Transfer	9.4	0.9–94.9	0.058	0.1	0.0–1.1	0.058
Negative (2 consecutive cultures)	9.8	2.7–34.7	0.001*	0.1	0.3–0.4	0.001
**Registration year**						
2010	Ref.	-	-	-	-	-
2011	2.5	0.4–16.9	0.339	0.4	0.1–2.6	0.339
2012	9.7	1.4–66.3	0.021*	0.1	0.0–0.7	0.021
2013	4.5	0.8–25.0	0.090	0.2	0.0–1.3	0.090
2014	4.9	0.8–29.1	0.078	0.2	0.0–1.2	0.078
2015	1.6	0.3–9.7	0.583	0.6	0.1–3.6	0.583
2016	2.6	0.4–15.5	0.288	0.3	0.1–2.3	0.288
2017	6.2	0.8–48.1	0.076	0.2	0.0–1.2	0.076
2018	8.6	1.1–66.2	0.038*	1.1	0.0–0.9	0.038
**Previous drug history**						
No	Ref.	-	-	-	-	-
Yes	0.5	0.1–2.1	0.347	1.6	0.6–4.1	0.347
**Previous drug history**						
New	0.0	0.0	0.999	0.5	0.1–2.1	0.987
PTR1	0.0	0.0	0.999	0.0	0.0	0.855
PTR2	Ref	-	-	-	-	-
**Patient category**						
New	Ref.	-	-	-	-	-
Other	1.2	0.1–26.2	0.930	0.9	0.0–19.8	0.930
Relapse	0.7	0.2–2.4	0.522	1.5	0.4–5.5	0.522
TF1	0.7	0.2–2.8	0.623	1.4	0.4–5.6	0.623
TF2	0.6	0.1–3.0	0.500	1.8	0.3–9.6	0.500
**Pulmonary or extra-pulmonary**						
Pulmonary	Ref.	-	-	-	-	-
Extra pulmonary	6 034 258	0.0	0.999	1.9	1.2–1.2	-
**Age group (years)**						
0–19	0.1	0.0	0.997	6.9	0.0	0.993
20–39	0.5	0.1–1.6	0.665	2.2	0.6–7.6	0.229
40–59	0.7	0.2–2.3	0.528	1.5	0.4–5.3	0.528
60	Ref.	-	-	-	-	-
**Gender**						
Male	Ref.	-	-	-	-	-
Female	2.1	1.1–3.9	0.021*	0.5	0.3–0.9	0.021

* , significant *p* ≤ 0.05.

Ref., Reference; COR, crude odd ratio; CI, confidence interval; AOR, adjusted odds ratio; *p* = 0.005; PT1, patient treatment regimen 1; PT2, patient treatment regimen 2; TF1, treatment failure after regimen 1; TF2, treatment failure after regimen 2.

## Discussion

The findings of the study report drug-resistant TB successful treatment outcome rate of 57.9%. The successful treatment outcome rate is the sum of 51.2% cure and 6.8% treatment completed rate. Drug-resistant TB treatment is associated with increased mortality and poor treatment outcome globally.^25,10,33^ The successful treatment outcome rate is higher than that of previous studies conducted in Abuja, Nigeria, which is 48.8%^34^ and elsewhere (Ethiopia, 28.9%) and Malaysia, 53.4%.^35^ The disparity could be attributable to the fact that the number of study participants in each study differs. The findings are lower than those reported in Cameroon, which reported 78.6% and in Northeast Ethiopia, which reported 80.7% overall successful treatment outcome.^6,21^

The results of this study had a cure rate of 51.2% higher than other studies conducted in South Africa: 28%, 41% and 51% reported in Eastern Cape, KwaZulu-Natal and Khayelitsha, respectively.^36,37,38^ The SA NDoH strategic plan set a target of 65% for MDR-TB success in 2018–2019.^39^ The 6.8% treatment completion rate of this study is higher than the findings of Jikijela,^36^ who reported 6.6%. However, Brust et al.^37^ reported a higher treatment completion rate of 16.5% than this study.^38^ The study cohort report 55.8% of male patients had drug-resistant TB than 44.2% of female patients. The findings are consistent with the findings from other studies, which report that men are more likely than women to be infected by TB or DR-TB.^40,41,42^ In the 2020 Global TB report, adult men carried the greatest burden of TB, accounting for 56% of all cases, compared with adult women’s 33% and children’s 11%, although TB can affect anyone irrespective of age or gender.^43^ The age group from 20 to 39 had the majority of patients than the other age group in this study. This suggested that the disease is prevalent in the most economically productive age group. Most of the patients were from the Waterberg district, which had 304 (79.0%) patients, whereas the other four districts had fewer patients in the Limpopo province. This is ascribable to the fact that the MDR-TB unit is in the Waterberg district at Modimolle. The Waterberg district has the mining industry, which provides mainstream employment for migrant and immigrant populations.^44^ Perhaps this is why the district was reported to have the highest TB incidence in the Limpopo province in 2015–2016.^30^

High cases of multidrug-resistant TB have previously been reported in South Africa.^28^ Table 2 shows the clinical characteristics of drug-resistant TB patients from 2010 to 2018. In this study, most patients (99.5%) were diagnosed with PTB than those with extra-PTB (0.5%). In the Eastern Cape, a high value of pulmonary MDR-TB was reported at 78%, while extra-pulmonary MDR-TB was 5.4%.^36^ Mabunda et al.^45^ conducted a study in the Limpopo province and reported a similar rate of PTB (88.5%) and extra-PTB (11.5%).

Out of the 385 TB patients in this study, most of the patients, 197 (51.2%) had been cured, 26 (6.8%) had completed treatment and 78 (20.6%) were lost to follow-up. The rate of loss to follow-up and death in this study may also influence the successful treatment outcome rate, as observed in other studies.^6^ A research study reported a treatment failure of 65.1% for MDR TB in the Eastern Cape.^36^ The study by Jikijela^36^ reported 65.1% treatment failure, which was attributed to death (44.3%), default (11.1%), treatment failure (4.8%) and transferred out (3%). The study by Cox et al.^38^ showed low death (14%), high defaults (32%) and failure of treatment of 3% were associated with unsuccessful treatment outcomes.^38^

Factors associated with drug-resistant TB successful treatment outcome in this study were negative sputum results after the 6-month interval, year of treatment initiation, and gender. In bacteriologically diagnosed PTB patients, sputum conversion is a critical predictor of treatment response and reduced infectivity. Sputum smear predicts cure and treatment success rates, two significant positive outcomes of TB treatment; sputum conversion is an important performance target for TB control efforts.^46,47,48^ Patients who were registered for treatment in 2012 and 2018 had a significant association with successful treatment in the study. The introduction of integration and decentralisation of TB and HIV health services, roll out of Gene Xpert MTB/RIF and other molecular diagnostic test technologies were introduced in 2010 in South Africa, which could have contributed to increased case detection and successful rate outcome.^23,49,50^ Types of drugs (mono) in this study contributed towards the treatment success rate. The current studies differ from Misra et al.,^51^ who reported that rifampicin-resistant TB and extensive drug-resistant TB (XDR-TB) contributed to successful treatment outcomes. This study’s age group of 20–39 was associated with successful treatment outcomes. Younger age, biological sex, comorbidity or underlying health issues, pre-treatment weight, and socioeconomic situation or family support have all been identified as influencing TB treatment outcomes in sub-Saharan Africa.^52,53^ The treatment of drug-resistant TB is difficult, time-consuming, and expensive. It necessitates the use of numerous anti-TB drugs that are more toxic and inefficient than the first-line therapies used for sensitive TB, which frequently leads to subpar therapeutic outcomes.^47^

Early diagnosis and effective treatment can prevent many TB-related morbidities, transmission and development of MDR-TB, but managing them is not always simple for various reasons.^8^ Monitoring the effectiveness of the DR-TB treatment programme critically depends on the evaluation of DR-TB treatment outcomes and subsequent investigation of factors associated with successful treatment outcomes and identifying high-risk and vulnerable populations, especially in a country such as South Africa, where both the TB and HIV epidemics are rampant.

### Strengths and limitations

To the author’s knowledge, this is the first study to evaluate drug-resistant TB treatment outcome, post-integration and decentralisation of TB and HIV services and roll out of Gene Xpert and other molecular techniques at the primary care level in the Limpopo province. Drug-resistant TB surveillance is crucial in well-informed decision-making, planning and resource allocation. At both the regional and national levels, epidemiological and public health research is essential, and it would be beneficial to incorporate the findings into national TB control plans.

The study used secondary data, other variables such as sociocultural (stigma, cultural views, education), economic employment, occupation, income, and personal behavioural factors (smoking, alcohol use and abuse); aspects that affect the patient treatment outcome were not recorded. Improving tracking and monitoring of patients transferred to other health facilities may improve the exclusion of incomplete medical records.

### Recommendations

The study recommends using provincial DR-TB surveillance data to best prioritise intervention strategies that will impact the estimated future burden of drug-resistant TB disease to achieve End TB targets. Increased community awareness of DR-TB, suggestive signs and symptoms to promote health-seeking behaviour and active TB case finding should be seriously considered. Equipping healthcare facilities with rapid diagnostic tests and building the capacity of healthcare providers to diagnose DR-TB may help to reduce time delays in DR-TB diagnosis and treatment initiation. Specific interventions such as early diagnosis, early treatment initiation and patient treatment monitoring may further improve the treatment success rates in the Limpopo province.

## Conclusion

In conclusion, DR-TB successful treatment outcome rate in this study is lower than national targets and targets recommended by WHO. The findings reveal that district and provincial TB control programmes need to focus on targeted intervention techniques that improve treatment outcomes.
